# Neighborhood Social Capital in Relation to Late HIV Diagnosis, Linkage to HIV Care, and HIV Care Engagement

**DOI:** 10.1007/s10461-016-1581-9

**Published:** 2016-10-17

**Authors:** Yusuf Ransome, Ichiro Kawachi, Lorraine T. Dean

**Affiliations:** 1 Department of Social and Behavioral Sciences, Harvard T.H. Chan School of Public Health, 677 Huntington Avenue, Kresge 7th Floor, Boston, MA 02115 USA; 2 Department of Epidemiology, Johns Hopkins Bloomberg School of Public Health, Baltimore, MD USA

**Keywords:** Social capital, Philadelphia, USA, Late HIV diagnosis, Neighborhood, HIV care engagement

## Abstract

High neighborhood social capital could facilitate earlier diagnosis of HIV and higher rates of linkage and HIV care engagement. Multivariate analysis was used to examine whether social capital (social cohesion, social participation, and collective engagement) in 2004/2006 was associated with lower 5-year average (2007–2011) prevalence of (a) late HIV diagnosis, (b) linked to HIV care, and (c) engaged in HIV care within Philadelphia, PA, United States. Census tracts (N = 332). Higher average neighborhood social participation was associated with higher prevalence of late HIV diagnosis (b = 1.37, se = 0.32, p < 0.001), linked to HIV care (b = 1.13, se = 0.20, p < 0.001) and lower prevalence of engaged in HIV care (b = −1.16, se = 0.30, p < 0.001). Higher collective engagement was associated with lower prevalence of linked to HIV care (b = −0.62, se = 0.32, p < 0.05).The findings of different directions of associations among social capital indicators and HIV-related outcomes underscore the need for more nuanced research on the topic that include longitudinal assessment across key populations.

## Resumen

Barrio alto de capital social podría facilitar el diagnóstico precoz del VIH y mayores tasas de vinculación y el compromiso de la atención del VIH. Se utilizó un análisis multivariado para examinar si capital social (cohesión social, la participación social y el compromiso colectivo) en 2004/2006 se asoció con una menor prevalencia promedio de 5 años (2007–2011) de (a) un diagnóstico tardío del VIH, (b) vinculado a la atención del VIH, y (c) que participan en la atención del VIH en Filadelfia, PA, Estados Unidos secciones censales (N = 332). Mayor participación social nota promedio se asoció con una mayor prevalencia de diagnóstico tardío del VIH (b = 1.37, SE = 0,32, p < 0,001), vinculado a la atención del VIH (b = 1.13, SE = 0,20, p < 0,001) y menor prevalencia de la dedicada a la atención del VIH (b = −1,16, SE = 0,30, p < 0,001). Compromiso colectivo se asoció con una menor prevalencia de vinculado a la atención del VIH (b = -0,62; SE = 0,32, p < 0,05). Los resultados de diferentes direcciones de las asociaciones entre los indicadores de capital social y los resultados relacionados con el VIH ponen de relieve la necesidad de una mayor matizada investigación sobre el tema que incluye evaluación longitudinal a través de poblaciones clave.

## Introduction

On average, 24 % of persons newly diagnosed with HIV in the United States (U.S) receive a late HIV diagnosis, defined as being concurrently diagnosed with AIDS within 3 months of an initial diagnosis [1, 2]. Among the 1.2 million people living with HIV in the U.S., only 40 % were engaged in HIV care and 37 % prescribed anti-retroviral treatment [3]. Persons diagnosed with HIV late have lower likelihood of survival [4, 5] due to missing critical opportunities to fully benefit from anti-retroviral therapy [6, 7] that could reduce onward HIV transmission [8, 9]. Improving population rates of linkage to, and sustained engagement in HIV care, is important to reduce virologic suppression among individuals [10] and reduce HIV burden in the community [11].

HIV outcomes in the population, including late HIV diagnosis, linkage to HIV care and engagement in HIV care are driven by social and structural factors that include racial residential segregation, concentrated poverty, and social and human capital investments at the ecological level [12–16]. Therefore, addressing social and structural factors could significantly improve HIV prevention above individually-based biomedical and behavioral factors [17–19]. For instance, a recent economic evaluation of HIV prevention programs in Ontario, Canada found that province-wide community-based interventions that included increasing social support among residents, providing supportive housing, distributing condoms and needles and running anti-stigma campaigns, were associated with preventing 16,1672 new HIV infections, and saved the health care system approximately 6.5 billion dollars over 23 years [20].

Social capital can potentially be leveraged for HIV prevention interventions within communities [21–23]. Social capital is defined differently according to three theoretical perspectives dominant in the social science and public health literature, each of which suggests a set of theory-based social capital indicators. James Coleman defined social capital as the function of social structures that facilitate actions, obligations and expectations among actors and organizations that make it possible to achieve ends [24]. Indicators include trust of others, as well as institutions, and ability to sanction deviance and enforce social norms [24, 25]. Pierre Bourdieu defined social capital as the aggregate of actual or potential resources linked to a durable network of institutionalized relations of mutual recognition and acquaintance [26]. Indicators include aggregate social support, collective social order, and participation in community organizations or other collective activities [26, 27]. Robert Putnam defines social capital as features of social organization that improve efficiency of society by facilitating coordinated actions [28]. Indicators include membership in civic and social organizations, generalized trust, and trust of one’s neighbor [29], which overlap and expand on the previous indicators. Moreover, social capital can be broadly defined as the structure of networks and collective resources within a community that individuals within that community can draw upon and benefit from [30, 31].

Each indicator of social capital may positively or negatively affect health and HIV outcomes in distinct or overlapping ways, therefore each are important to analyze separately. For instance, individuals in socially cohesive communities characterized by high trust and feelings of belongingness may project social norms that HIV infection is the result of promiscuous behavior, and may stigmatize individuals living with HIV who then may be less likely to seek HIV care and prevention resources [32, 33]. On the other hand, socially cohesive communities could foster a supportive environment for people to seek and utilize HIV testing [32].

Next, communities with high HIV prevalence but characterized by high coordinated and collective action, and high obligations and expectations of others may have higher prevalence of HIV care engagement. For instance, if an HIV testing and treatment center was to close in the neighborhood, but residents collectively agreed that this closure would make it difficult for HIV positive residents to obtain care, other residents (positive and HIV negative) could lobby to keep the center open.

Communities characterized by high resident participation in civic and social organizations (e.g., church and political groups) may also have lower late HIV diagnosis and higher HIV care engagement because of the potential for information and resource exchanges between HIV positive and negative individuals. Alternatively, higher community-level civic and social participation may be correlated with higher rates of late HIV diagnosis if participation is reflecting sero-converters who are getting tested as a function of the social support generated from participating in organizations that offer HIV testing. Relatedly, high HIV and related stigmas (e.g., of injection drug users) even within civically-engaged or cohesive communities may act as a barrier for neighbors engaging with one another to learn about HIV services [34]. Thus, HIV positive individuals may adopt an avoidance ritual [35] by deliberately seeking advice about HIV testing and actual HIV care outside of their community.

With respect to HIV outcomes, social capital is associated empirically with lower rates of infectious diseases [36], and HIV incidence and prevalence [37], through mechanisms such as higher awareness, knowledge and information sharing [38–40], HIV testing [41, 42], HIV disclosure [43], as well as lower HIV stigma and discrimination [42, 44, 45], and lower individual level risk behaviors that include infrequent condom use, and multiple sexual partners [37, 46–48].

Despite the theoretical links between social capital and HIV and the empirical evidence from international studies, there remains a paucity of published manuscripts on the topic in the U.S. Moreover, among the present studies, few have examined multiple social capital indicators in association with multiple HIV outcomes along the HIV care continuum (e.g., diagnosis, and care engagement) [49]. This analysis therefore investigates whether social capital is associated at the ecological level with prevalence of late HIV diagnosis, linked to HIV care, and engaged in HIV care in a large urban U.S. city. Results potentially can inform the state of HIV care engagement at both the local and national level.

## Methods

The city of Philadelphia, Pennsylvania (PA) was the setting for this study. It is the fifth most populous city in the U.S. [50]. Published data from year 2013 showed that the prevalence of late HIV diagnosis (defined here as an AIDS diagnosis within 12 months of being newly diagnosed) was 25.5 %, which is on par with the national average for that period [51]. Data on neighborhood social capital were available for 332 out of 381 Census tracts, based Census 2000 boundaries. The Census tract is a very small geographic scale and valid neighborhood unit to study social capital and health [52], and HIV outcomes [53].

### Measures

HIV surveillance data on the prevalence of persons with late HIV diagnosis, linked to HIV care, and engaged in HIV care at the ZIP code level were retrieved from HIVcontinuum.org, which is a web-database that contains several HIV outcomes from local health departments across five cities with high HIV burden [54]. HIV surveillance data were provided to HIVContinuum.org by the Philadelphia Department of Public Health, AIDS Activities Coordinating Office. These data contain the population-based sources of complete HIV infection and care engagement data reported as of 12/31/2012. Cases missing address or ZIP code at HIV diagnosis are excluded and cases diagnosed in a correctional facility were assigned to the ZIP code of the facility. For this study, only aggregate prevalence data and not actual count of cases were available.

*Late HIV diagnoses* represents the 5-year average (2007–2011) prevalence of adults/adolescents with an AIDS diagnosis within three months of newly diagnosed HIV.

*Linked to HIV care* represents the 5-year average (2007–2011) prevalence of adults/adolescents newly diagnosed with HIV with a reported CD4/viral load within 3 months of HIV diagnosis.

*Engaged in HIV care* represents the 5-year average (2007–2011) prevalence of adults/adolescents newly diagnosed with HIV from 2007–2011 and linked to HIV care with a reported CD4/viral load in year 2012.

Social capital data were retrieved from the Southeastern Pennsylvania Household Health Survey (SPHHS) administered by the Public Health Management Corporation [55]. Survey years 2004 and 2006 were combined, which were the only 2 years with social capital measures before the HIV exposure data. The SPHHS is a Random Digit Dialing household telephone survey of health, social, and behavioral items asked of persons 18 years of age and older across the five major counties of greater Philadelphia area. In 2004, the survey achieved a Philadelphia sample of 4415 with 27 % response rate, and in 2006, a sample of 4193 with a 24 % response rate. The characteristics of the sample across survey waves are intentionally similar and so combining the data was not a threat to temporal variability. The SPHHS response rate falls within the range other well-used and respected community surveys that use random-digit dialing [56–58] and that were issued during that time. As an additional strength, unlike some other community-based surveys, SPHHS includes cellular phone users, which minimizes selection bias associated with random-digit dialing techniques. There was an average of 30 respondents in each tract [inter-quartile range (IQR) = 21–39]. Individuals had fairly stable residence patterns with 16 years being the average length of residence in the community [IQR = 3–27]. There were five questions in the survey that capture social capital based on definitions corresponding to the three theoretical definitions described in the introduction. All the derived measures are validated based on face, convergent, and nomological validity criteria set forth by Lee and Kim [59].

*Social cohesion* was an indicator we created that aligns with definitions put forth by Robert Putnam. It was assessed by the following questions: Please tell me if you strongly agree, agree, disagree, or strongly disagree with the following statement: (1) I feel that I belong and am a part of my neighborhood, (2) most people in my neighborhood can be trusted. The third question was (3) please rate how likely people in your neighborhood are willing to help their neighbors with routine activities such as picking up their trash cans, or helping to shovel snow. Would you say that most people in your neighborhood are always, often, sometimes, rarely, or never willing to help their neighbor?. Prior research that used these data [52] suggested an oblique (promax) rotated principal components analysis (PCA), which demonstrated high reliability across these items (alpha = 0.76).

To obtain Census tract averages for social cohesion, a multivariate regression model was used with the PCA scores as the outcome and covariates individual’s age, sex, marital status, education, income, ratings of community, and rental or home ownership status. Empirical Bayes predicted values were estimated from the regression models, which produces aggregated scores removed of potential residual confounding from individual-level characteristics [60, 61]. The predictions were then mean aggregated to the Census tract. Characteristics of the individual level sample used to create this aggregate measure are available as an appendix table.

*Social participation* is an indicator that underpins both Putnam’s and Coleman’s definitions of social capital. This is a single item that queries individual’s participation in both civic and social organizations, based on the question: how many local groups or organizations in your neighborhood do you currently participate in such as social, political, religious, school-related, or athletic organizations? The measure for this study is a predicted count of individuals responses derived from negative binomial regression adjusted for the same covariates used to predict social cohesion. The predictions were then mean aggregated to the Census tract.

*Collective engagement* is based on Coleman’s definition of social capital about facilitating coordinated action to achieve certain ends. One item was available, which corresponded to the question, have people in your neighborhood ever worked together to improve the neighborhood? The measure for this study is a predicted probability of responding *yes,* and derived from logistic regression adjusted same covariates used to predict social cohesion. The predicted counts were then mean aggregated to the Census tract.

All social capital variables were aggregated to the Census tract using balancing weights provided in SPHHS, which accounts for the survey design by adjusting for sampling bias.

*HIV testing and HIV treatment center accessibility.* Increasing access to HIV testing and treatment are recommended for reducing late HIV diagnosis and improving linkage to and engagement in HIV care [62–64]. A list of HIV testing sites was generated by searching the National HIV and STD Testing web database [65] and a geographic database with locations of Ryan White HIV treatment centers across Philadelphia, PA (N = 39) available from OpenDataPhilly.org [66]. Leadership of the Philadelphia AIDS Activities Coordinating Office validated which centers were present before year 2007. This corresponded to a final list of N = 75 centers after removing duplicates. Access to HIV testing and treatment is defined as the nearest distance (in miles) from the centroid of each Census tract to the closest HIV testing and treatment facility, as calculated by the *Near Analysis Tool* [67] in ArcGIS Desktop 10.2 [68]. Social capital may be associated with HIV testing in adjacent neighborhoods and individuals may receive HIV testing in neighborhoods adjacent to their residence. To account for this possibility, a spatially lagged HIV testing variable was derived based on the mean distance of HIV testing from centroids in adjacent neighborhoods. The variable was calculated as above, but incorporated adjacent tracts by using a Queen contiguity-based spatial weights matrix created in GeoDa software [69].

*Assault rate* was included as a covariate. Crime—one element of social disorder is associated with psychological distress that is linked to HIV risk behaviors [22]. Social capital is negatively associated with crime [70, 71] but it has been insufficiently researched with respect to HIV-related outcomes including HIV testing and linkage to HIV care [72–74]. Crime data were provided by the Philadelphia Police Department and made available through OpenDataPhilly.org [66]. The measure is the 5-year average rate (2007–2011) of all types of assault per 1000 Census 2010 population. Assault rates were log-transformed to address its right-skewed distribution.

The following socioeconomic and demographic covariates associated with late HIV and other HIV-related outcomes [75–78] were included: percent of black/African American residents, percent of males, percent of persons 25 years and older with less than a 9th grade education, percent of persons 16 years and older unemployed, median income, and percent of persons living in poverty within the Census tract. These data were retrieved from the Census 2000 estimates, Summary Files 3 Demographic Profiles 2 and 3 [79].

### Statistical Methods and Analysis

#### Areal Interpolation

HIV surveillance data at the Census tract level were derived in two steps using the *areal interpolation* function in ArcGIS 10.2 software [67], which is a kriging-based method to smooth data across different spatial aggregation units and across units missing data [80]. First, using the HIV surveillance data at the ZIP code level, estimates for ZIP codes missing data were interpolated for each outcome separately (missing, N = 6/45 for late HIV diagnosis; N = 1/45 for linked to HIV care; and N = 2/45 for engaged in HIV care). The areal interpolation allowed one covariate, and income inequality, as measured with the GINI coefficient from the American Community Survey 5-year (2007–2011) estimates was selected because of prior research documenting an association with late HIV diagnosis [81]. Areal interpolation was employed with the following parameters: covariance semivariogram model; lag distance of 1000 meters; and search neighborhood parameters (maximum and minimum of four neighbors). Using those aforementioned parameters across each of the three HIV outcomes; in an iterative process, lag size and variogram model type were manipulated, separately for each outcome, to improve the fit and validity of the prediction model. For the late HIV diagnosis outcome, the predicted data fit best when the model type was “K-Bessel” and the number of lags was 15 and all other inputs were set to default. For the linked to HIV care and engaged in HIV care outcomes, the predicted data fit best when the model type was “Spherical” and the number of lags was 12, and all other inputs were set to default. Areal interpolation produces a smoothed surface map. In the second step, each smoothed surface map for the separate HIV outcomes was used to predict data to the Census tracts using the areal interpolation *layer to polygons* function [82].

#### Descriptive Analysis

Pearson correlations at the Census tract level were calculated to examine the associations among study covariates. Then the median prevalence and interquartile range for the three HIV outcomes across Census tracts were estimated. Next, choropleth maps were created in ArcGIS 10.2 of the prevalence of the HIV outcome data at the original level (i.e., ZIP codes) and the smoothed predicted estimates at the Census tract level with the locations of HIV testing and treatment centers overlaid. Mean social capital was mapped at the Census tract for the HIV outcomes and z-scores for the social capital variables because each indicator was measured originally on different scales.

Moran’s *I* estimated the degree of spatial clustering for the HIV outcomes at the original ZIP code level and social capital at the Census tract level using a spatial weights matrix with nearest neighbor of k = 4 for ZIP codes, and k = 7 for Census tracts in the calculation. Moran’s *I* evaluates whether a pattern observed is clustered, dispersed, or random. A statistically significant value positive Moran’s *I* indicates that high values (i.e., rates or prevalence) spatially cluster near other high values, and that pattern is not random [83]. Last, Mann–Whitney *U* tests were used to determine whether excluded Census tracts (N = 49) were different from those included (N = 332) on neighborhood unemployment, education, median income, and poverty level. Significance was assessed at alpha *p* < 0.05.

#### Multivariate Multivariable Analysis

Multivariable analysis was performed in two steps. First, to determine the extent of spatial autocorrelation, spatial regression was run in GeoDa 1.01 software [69] for each outcome separately with social capital predictors, socioeconomic covariates and assault rate. Spatial regression models used a weights matrix for the Census tract with (k = 7) nearest neighbors. Regression diagnostics helped determine which type of model significantly reduced any autocorrelation found. For late HIV diagnosis, an error model was determined best, and a spatial lag model for linked to HIV care and for engaged in HIV care. These models for each outcome were re-estimated with queen contiguity symmetrical weights. The predicted residuals for each outcome were used in the multivariable model corresponding to that outcome, but not displayed in results table because they have no interpretation.

Generalized structural equation (GSEM) models were generated in Stata 14.1 [84] to estimate a multivariate model where the three HIV outcomes were simultaneously predicted by the social capital variables and all covariates. Using a multivariate model allowed us to directly test whether the magnitude of social capital associations are equivalent across the three HIV outcomes. The guiding hypothesis was that social capital will have a larger impact on late HIV diagnosis than the other two outcomes. Specifically, social capital was expected to be more strongly related to late HIV diagnosis given that diagnosis is furthest upstream on the HIV care continuum. Therefore, social capital would be expected to have weaker associations on the linked to HIV care and engaged in HIV care outcomes, which are further downstream on the HIV continuum.

## Results

### Spatial Interpolation Predicting Census Tract HIV Estimates

**Fig. 1:**
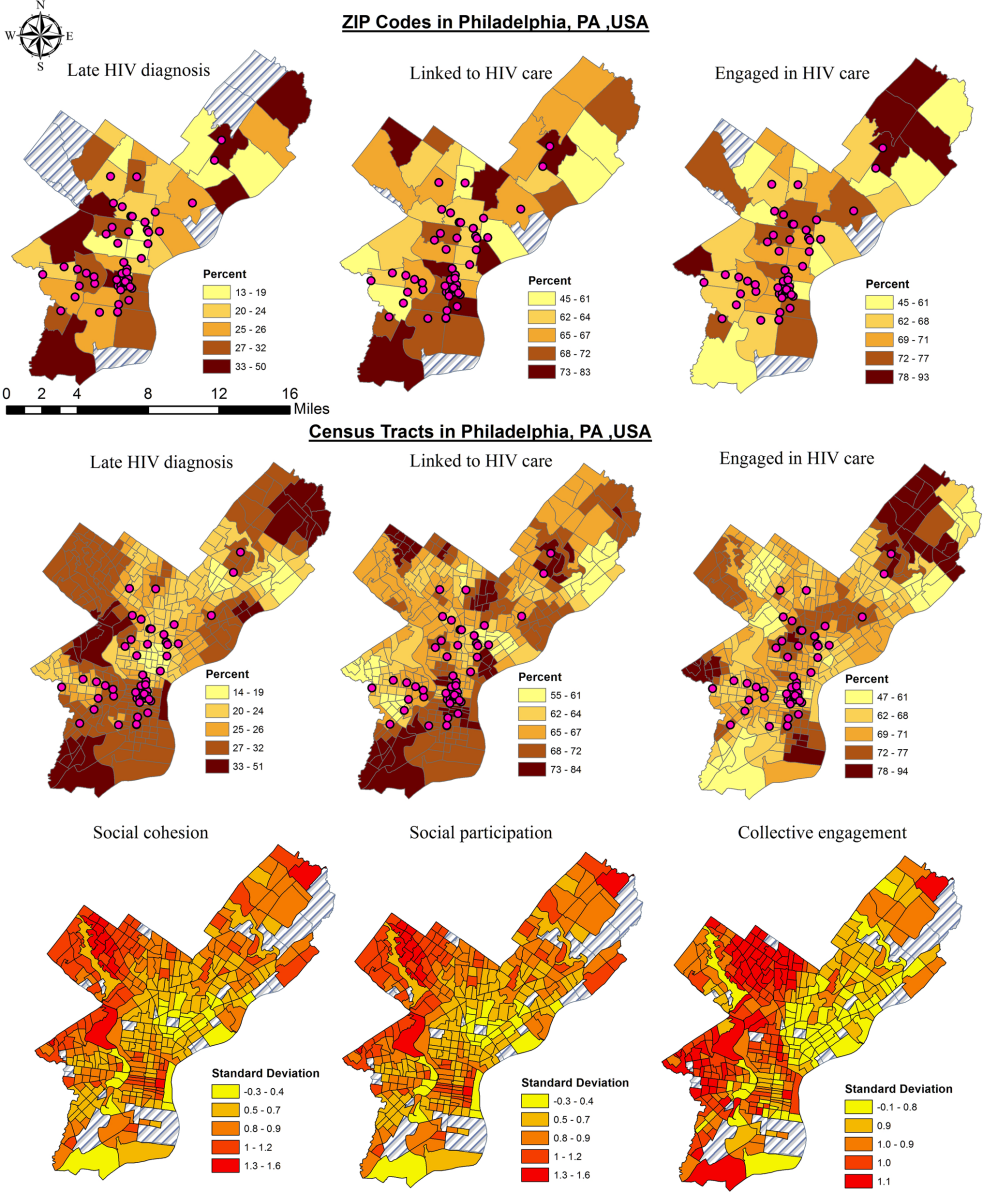
*Top row* are original data at the ZIP code level (N = 45) Philadelphia, PA, for 5-year average (2007–2011) HIV prevalence data from HIVcontinuum.org. *Second row* contains the areal interpolated data at the Census tract level (N = 332). *Third row* contains social capital data at the Census tract level, Philadelphia, PA for year 2004/2006. *Lighter color* represents greater presence for the exposures and outcomes.  *Shaded regions* are areas where data were not originally available. *Filled circles* represents HIV testing and HIV treatment centers (N = 75), some locations are close and overlap, so not all points are visible (Color figure online)

Adequate fit and validity of the areal interpolation model is assessed by how close the mean-squared-standardized (RMSS) is to 1 and how similar the mean-squared (RMS) is to the average standard error (ASE). The fit of the ZIP code to Census tract estimates for late HIV diagnosis had RMSS value of 1.2, RMS of 7.8 and ASE of 7.5. For linked to HIV care, RMSS was 1.03, and RMS was 6.5 and ASE was 8.1. For engaged in HIV care, RMSS was 1.17 and RMS was 9.4 and ASE was 10.4 (results not displayed). Visual inspection of choropleth maps of the predicted surface for the Census tracts corroborated high consistency with the patterns observed for the data at the original ZIP code level (Fig. 1).

### Descriptive Associations

Social cohesion had significant moderate positive correlation with social participation (r = 0.69, p < 0.01) and collective engagement (r = 0.49, p < 0.01), and collective engagement had a large and positive correlation with social participation (r = 0.61, p < 0.01). All social capital variables were negatively correlated with poverty and education. The median prevalence of late HIV diagnosis within 3 months of an initial HIV infection for 2007–2011 was 26 % (inter-quartile range (IQR) 23–28 %); medial prevalence of linkage to HIV care was 66 %, (IQR = 64–69 %) and median prevalence of engagement in HIV care was 69 %, (IQR = 66–71 %) (results not displayed). Late HIV diagnosis was significantly correlated with social cohesion (r = 0.15, p < 0.01) and social participation (r = 0.27, p < 0.01). Engagement in HIV care was inversely correlated with social participation and collective efficacy (r = − 0.12, p < 0.05). Distance to HIV testing center or treatment center was positively correlated with late HIV diagnosis (r = 0.33, p < 0.01), and linked to HIV care (r = 0.15, p < 0.05). Lagged HIV testing center was moderately correlated with linked to HIV care (r = 0.34, p < 0.01) (Table 1).

**Table 1:** Pearson product moment correlation coefficients among study variables

		1	2	3	4	5	6	7	8	9	10	11	12	13	14	15
1	Social cohesion^a^	1														
2	Social participation^b^	0.69**	1													
3	Collective engagement^c^	0.49**	0.61**	1												
4	Late HIV diagnosis	0.15**	0.27**	0.08	1											
5	Linked to HIV care	0.03	0.08	−0.09	0.34*	1										
6	Engaged in HIV care	−0.04	−0.12*	−0.12*	−0.10	0.07	1									
7	Distance to HIV testing or treatment center	0.37**	0.43**	0.11*	0.33*	0.15**	0.13*	1								
8	LAG Distance to HIV testing or treatment center	0.43**	0.45**	0.12*	0.34**	0.12*	0.13*	0.86**	1							
9	Assault rate per 1000 capita	−0.27**	−0.40**	−0.08	0.28*	−0.15**	0.02	−0.36**	−0.35**	1						
10	Percent black	−0.44**	−0.13*	0.57**	−0.17**	−0.21**	−0.03	−0.31**	−0.33**	0.28**	1					
11	Percent male	0.05	−0.09	−0.19**	0.04	0.06	−0.13*	0.02	−0.07	0.02	−0.26**	1				
12	Percent 25 older with <9th grade education	−0.13*	−0.37**	−0.23**	−0.25**	−0.01	0.20**	−0.20**	−0.19**	0.11*	0.01	−0.06	1			
13	Percent 16 older unemployed	−0.32**	−0.35**	0.04	−0.23**	−0.18**	−0.02	−0.45**	−0.46**	0.32**	0.47**	−0.10	0.23**	1		
14	Median income	0.50**	0.59**	0.20**	0.20**	0.15**	−0.06	0.50**	0.49**	−0.39**	0.00	0.00	−0.34**	−0.45**	1	
15	Percent in poverty	−0.63**	−0.62**	−0.19**	−0.32**	−0.13*	0.07	−0.52**	−0.49**	0.42**	−0.07	−0.08	0.57**	0.65**	−0.67**	1

* *p* < 0.05

** *p* < 0.01. Two-tailed tests

^a^Social cohesion: Census tract regression score aggregate from a principal components index of trust, neighborliness and belongingness. Higher scores correspond to higher social cohesion

^b^Social participation: Census tract mean aggregate of predicted count of individual’s participation in social, political, religious or other organizations in neighborhood. Higher scores correspond to higher social participation

^c^Collective engagement: Census tract mean aggregate predicted response to (yes) people worked together to improve the neighborhood

The Moran’s *I* coefficients correspond to maps in Fig. 1. No significant clustering was observed for late HIV diagnosis (*I* = −0.04, p = 0.41), linked to HIV care (*I* = −0.07, p = 0.30) or engaged in HIV care (*I* = 0.02, p = 0.31) at the ZIP code level. Significant clustering was observed at the Census tract level for all three HIV outcomes: late diagnosis (*I* = 0.70, p < 0.002), linkage to (*I* = 0.64, p < 0.002) and engagement in care (*I* = 0.55, p < 0.002), and for social cohesion (*I* = −0.28, p < 0.002), social participation (*I* = 0.53, p < 0.002), and collective efficacy (*I* = 0.48, p < 0.002).

There were no differences in unemployment, education, median income or poverty between Census tracts included (N = 332) and not included in the study (N = 49) (results not displayed).

### Multivariate Association Among Social Capital and HIV Outcomes

In multivariate analysis, the associations between social capital and the HIV outcomes are adjusted for HIV testing and treatment center, assault rate, and Census-tract sociodemographic and economic characteristics (hereafter, covariates) (Table 2). Social cohesion was not statistically associated with any of the HIV outcomes adjusting for covariates. A 1 standard deviation (SD) higher mean aggregate social participation was associated with slightly 1 % higher prevalence of late HIV diagnosis (b = 1.37, se = 0.32, p < 0.001), persons linked to HIV care (b = 1.13, se = 0.20, p < 0.001), and lower prevalence of persons engaged in HIV care (b = −1.16, se = 0.30, p < 0.001), adjusted for covariates. Collective engagement was statistically associated with 0.62 % higher prevalence of persons engaged in HIV care (b = 0.62, se = 0.27, p < 0.05), adjusted for covariates.

**Table 2:** Association between social capital and selected HIV outcomes across the HIV treatment cascade in Philadelphia neighborhoods (N = 332 Census tracts)

Mean (SD) of each variable^†^	Late HIV diagnosis b (se)	Linked to HIV care b (se)	Engaged in HIV care b (se)
Social cohesion^a,d^, 9.28 (0.76)	− 0.45 (0.37)	−0.43 (0.31)	0.16 (0.36)
Social participation^b,d^, 0.79 (0.20)	1.37 (0.32)***	1.13 (0.20)***	−1.16 (0.30)***
Collective engagement^c,d^, 0.65 (0.07)	−0.63 (0.38)	−0.62 (0.32)*	−0.01 (0.36)
Distance (in miles) to nearest HIV testing center or treatment facility, 1.14 (0.97)	−0.10 (0.28)	−0.12 (0.24)	0.62 (0.27)*
LAG Distance (in miles) to nearest HIV testing center or treatment facility^e^, 1.07 (0.72)	0.96 (0.29)**	0.30 (0.24)	0.42 (0.27)
Assault rate per 1000 capita, 37.01 (36.71)	−0.03 (0.18)	−0.12 (0.15)	−0.08 (0.02)**
Percent black, 45.22 (37.05)	0.09 (0.31)	−0.30 (0.26)	−0.05 (0.30)
Percent male, 46.76 (5.73)	0.00 (0.20)	0.11 (0.17)	0.04 (0.20)
Percent 25 older with <9th grade education, 7.50 (6.43)	−1.07 (0.22)***	0.14 (0.18)	0.94 (0.21)***
Percent 16 older unemployed, 6.05 (3.07)	−0.06 (0.20)	−0.48 (0.17)**	−0.30 (0.19)
Median income, $32,291 ($18,882)	−0.38 (0.22)	−0.06 (0.18)	−0.21 (0.21)
Percent in poverty, 19.11 (14.75)	0.09 (0.28)	0.47 (0.24)	−0.13 (0.27)

*b* beta coefficient, *SE* standard error

* *p* < 0.05

** *p* < 0.01

*** *p* < 0.001

^†^All predictors have been z-scored transformed to a mean of 0 and standard deviation (SD) of 1, for the multivariable analysis

^a^Social cohesion: Census tract regression score aggregate from a principal components index of trust, neighborliness and belongingness

^b^Social participation: Census tract mean aggregate of predicted count of individual’s participation in social, political, religious or other organizations in neighborhood

^c^Collective engagement: Census tract mean aggregate predicted response to (yes) people worked together to improve the neighborhood

^d^Coded such that it corresponds to higher social capital

^e^LAG are for distance of the adjacent neighbors based on Queen Contiguity weights matrix at the Census tract level

Social participation was the only significant social capital indicator across all three HIV outcomes. We therefore focused our hypothesis test of higher magnitude of association for late HIV diagnosis vs the other HIV outcomes, to this indicator only. The magnitude [absolute value] of association between social participation on late HIV diagnosis (b = 1.37) was not statistically larger than the magnitude of the association with linked to HIV care (b = 1.13) χ^2^ = 0.31, p = 0.58, nor engaged in HIV care (b = 1.16) χ^2^ = 0.21, p = 0.64 (results not displayed).

Among the covariates, 1 SD in distance to adjacent testing centers was associated with a 1 % higher prevalence of late HIV diagnosis (b = 0.96, se = 0.29, p < 0.05) but not significantly associated with any other HIV outcome. A 1 SD increase in assault rate was marginally associated with lower prevalence of engaged in HIV care. Poverty and median income had no statistically independent associations with any of the HIV outcomes, adjusting for other covariates.

## Discussion

This is the first study to examine multiple social capital indicators in relation to multiple HIV outcomes along the HIV care continuum in the U.S. Results indicate mixed evidence regarding the direction of association social capital should theoretically have on HIV outcomes. This ecological analysis of Census tracts in Philadelphia found no evidence that social cohesion was statistically related to late HIV diagnosis, although the association is the expected direction as found in a recent ZIP-code level ecological study conducted in New York City by Ransome, Galea, and Pabayo et al. [85]. That NYC-based study, however, differed from this study in several ways. First, while both this and the NYC-based study’s definition of social cohesion includes a component of trust and neighbors’ willingness to help, the third component differs. The social cohesion index in this study included a measure of belongingness while the NYC-based study asked about perceived close-knit relationships. The NYC-based also examined social capital among men and women, which contrasts with this study’s use of aggregate data for the entire HIV infected population.

This study’s findings indicate that each of the social capital indicators was positively correlated with HIV testing, and social cohesion was negatively correlated with crime. These directions are consistent with associations reported in prior research [41, 42, 71]. However, findings in this study cannot preclude reverse causality given the cross-sectional nature of these data.

The present study’s results show that higher social participation was associated with higher late HIV diagnosis. The direction found in this study is similar to the direction found by Ransome, Galea and Pabayo et al. among men [85], measured by a civic engagement indicator that includes a component of participating in local organizations, just as in this study. While in general, social capital would be expected to be associated with lower late HIV diagnosis, the directions found in these two studies may indicate a complex pattern more detailed data are required to answer. For instance, in both ours and the NYC-based study, information on the type of organizations where individuals participated was not assessed. Social capital generated across different types of organizational participation matters for HIV outcomes.

It is therefore plausible that the positive association between social participation/civic engagement and late HIV diagnosis could reflect participation in different type of organizations and potentially higher membership in organizations with high HIV testing norms. For instance, Campbell, Williams and Gilgen study of social capital and HIV in South Africa found that for men, participation in *stokvels* (savings club with social activities) was associated with higher likelihood of individuals being HIV+ , but participation in sports clubs were associated with lower likelihood of members being HIV+ [86].

The present study may be the first to document that social capital is empirically associated with prevalence of persons linked to HIV care and engagement of HIV care in the community. Consistent with what theory would predict; higher social participation was associated with higher prevalence of persons linked to HIV care. The negative association between social participation and prevalence of persons engaged in HIV care could reflect a diminishing return of social capital’s association on outcomes very close along the HIV care cascade. Specifically, high social capital may facilitate higher linkage to care within three months but may those benefits may diminish between 4 and 12 months—the operational time period that distinguishes between linked and engaged in HIV care.

Without longitudinal data on types of organizational participation, results are limited to speculation. One potential explanation for differences in direction of association is that community-level and individual-level psychosocial mechanisms such as information exchange and self-esteem and coping [32, 87, 88] facilitate protective associations on short-term behaviors such as diagnosis and being linked to care but not long-term behaviors. Next, it is plausible that social capital may become disruptive [89] in the long term and associated with HIV risk behaviors that encompass the dark side or negative aspects of social capital [90]. It is also possible that social participation may have changed. For instance, social participation during the 9 months between being linked to HIV care and engaged in HIV care could have changed from organizations characterized by HIV prevention to organizations characterized by HIV risk and delinquency.

Others have noted that social capital may not benefit all groups [91] and consequently may not be associated with lower HIV risk [92]. For instance, while religious institutions has been a space where many blacks traditionally have drawn social capital from [93], this same space has been a source of stigma for black men who have sex with men (MSM) [94]—the subpopulation with highest rates of incidence and late HIV among blacks [95].

There was limited support that collective engagement was associated lower prevalence of persons linked to HIV care in the Census tract. Again, these findings run contrary to what theory would predict. These findings may be related to limitations assessing social capital and HIV using cross-sectional data. For instance, in areas with high disease burden; high social capital may be the result of community members becoming more collectively engaged as a way to draw attention to its needs and secure health resources, which has been found in other chronic disease studies in Philadelphia, PA [52, 96, 97]. It is possible therefore that rates of linkage to HIV care were low in the past and that motivated community members to coalesce to resolve the problem, which showed up in cross-sectional studies as high social capital in high-prevalence areas.

Another possibility is that higher rates of collective engagement in community and social organizations reflect need by persons already afflicted with high HIV burden within impoverished communities [98]. For instance, one study found that food insecurity among people living with HIV/AIDS was associated with residence in neighborhoods with poverty and with poorer HIV treatment adherence [99]. These explanations could also potentially explain why collective engagement was associated with lower linkage to HIV care.

This study finds no evidence to support the hypothesis that social capital would have a larger magnitude of association on late HIV diagnosis given it is further upstream the HIV care continuum than linked to and engaged in HIV care. These findings potentially indicate that social capital could be leveraged as an HIV prevention strategy at any of these points along the HIV care continuum. One alternative explanation could be that the impact of social capital on HIV care engagement is mediated through an indirect impact of social capital on late HIV diagnosis. However, that inquiry was outside the scope of this study and would require temporal data on the outcomes and path mediation analysis.

The findings and meaning of our results should be considered in context of the following study limitations. While our social capital measures were valid indicators based on the social capital theory, the composition of our measures such as social cohesion and collective engagement do not correspond fully to other validated social capital scales used in the literature [100]. However, the availability of social capital measures differ across surveys, which is one limitation of social capital research [101, 102] in general. Nevertheless, the measures used in this study can help to build evidence in the literature with regard to the utility in social science research.

This study used a single-item measure we called collective engagement to reflect James Coleman’s definition of social capital as engagement among actors to achieve certain ends. Following recommendations on assessing the validity of social capital measures put forth by Lee and Kim [59], this measure shows strong face-validity given that the question wording directly asks about people working together. Second, the large (i.e., r = 0.49 and r = 0.61) correlations of collective engagement with the other two social capital measures suggests these are capturing the underlying phenomena of social capital. Third, the collective engagement measure demonstrates nomological validity because like social cohesion and social participation, it also has a negative correlation with violent crime—a direction expected and empirically demonstrated with social capital [59].

Social capital stratified by socio-economic or demographic subgroup or disaggregate types of social participation could not be assessed. The quantity and quality of social capital varies across strata such as social class [25, 103]. Therefore, subgroup differences in social capital may moderate the associations on health. For instance, Hutchinson, Putt and Dean et al. found that neighborhood racial composition moderated the association between social capital and mortality rates in Philadelphia, PA [104]. The association between social capital and health [105] and HIV [106] could be moderated by socio-economic and demographic characteristics of group membership especially for persons who are marginalized or excluded from membership via competition for resources [107–109].

Next, only publicly available HIV surveillance data at the aggregate level were available. Therefore, data could not be stratified by sex nor race nor transmission status to investigate potential differences across key population groups. Given the limited the ability to distinguish the types of social participation and no data by race and transmission group, we could not discriminate the potential divergent associations. For instance, it is possible that social participation from religious compared to secular organizations have different impacts on HIV for Black MSM groups across race and transmission status [85].

HIV prevalence data included cases diagnosed in correctional facilities and assigned to the ZIP code of the facility. Unfortunately, those cases could not be identified in these aggregated data and thus could not be removed. This potentially is a problem because the SPHHS study did not assess social capital or other measures among institutional populations. Persons diagnosed within correctional facilities will typically have consistent access to healthcare, which may mean that late HIV diagnosis rates and engagement in care could be artificially higher in ZIP codes with correctional facilities. Without disaggregated data, this analysis could not assess the degree of potential bias in associations reported.

The generalizability of study results to other U.S. settings or ecological levels is limited. Specifically, as seen with differences in spatial clustering of HIV at Census tract versus ZIP code; correlations and regression estimates will also vary across ecological units due to the modifiable unit areal unit problem (MAUP) [110]. However, this study did not re-aggregate data but rather smoothed the data over a continuous geographic surface, which mitigates some limitations within the MAUP [111]. Moreover, Census tract is a particularly relevant unit for studying social relationships at the ecological level [112] given lower heterogeneity and greater temporal spatial stability than ZIP codes [113]. Using HIV surveillance data at a very fine geographic level also enhances the opportunity for precise geographically targeted HIV prevention initiatives [114].

Some strengths of this analysis include building a complex database of HIV surveillance, Census, administrative, and household survey data to address the paucity of research on social capital and HIV in the U.S. The methods used to interpolate HIV data across geography demonstrate the feasibility of utilizing geospatial technology to enhance HIV prevention research using publicly available data. Next, these results contributed to the broader literature and advanced prior ecological studies by examining multiple social capital indicators across multiple HIV outcomes along the HIV care continuum. Additionally, the study validated the new measures of social capital through several criterion, which included face validity, convergent validity, nomological validity, and predictive validity [59].

## Conclusion

This study highlights that neighborhood social capital is ecologically associated with population level HIV/AIDS outcomes along the care cascade in a large urban U.S. city. Differences in which social capital indicators were significant along with varying directions of associations across social capital indicators and HIV outcomes highlight the complexity of this research. The results lay a foundation for future studies to assess the relationship between multiple dimensions of social capital and HIV outcomes using prospective study design, multilevel methods, and across the intersection of race and transmission group.
